# Seroprevalence of Louping Ill virus (LIV) antibodies in sheep submitted for post mortem examination in the North West of Ireland in 2011

**DOI:** 10.1186/2046-0481-65-20

**Published:** 2012-12-11

**Authors:** Damien Barrett, Daniel M Collins, Guy McGrath, Colm Ó Muireagain

**Affiliations:** 1 Department of Agriculture, Food and Marine, Sligo Regional Veterinary Laboratory, Doonally, Sligo, Ireland; 2 Centre for Veterinary Epidemiology and Risk Analysis, School of Veterinary Medicine, University College Dublin, Belfield, Dublin 4, Dublin, Ireland

**Keywords:** Louping Ill, Sheep, Routine post mortem, Prevalence, Ireland

## Abstract

Blood samples were collected opportunistically at routine post mortem examination from 199 sheep which came from 152 flocks. The location of each submitting flock was mapped. Sera were tested using a goose blood haemagglutination inhibition assay for louping ill virus. There was an animal level prevalence of 8.5%, and a flock level prevalence of 9.8%. The greatest proportion of seropositive animals was identified among the animals older than 24 months of age. The elevation of the land associated with positive flocks was greater than that of negative flocks. Lesions of non-suppurative meningoencephalitis were observed in three of the 199 animals.

## Background

Louping ill is a tick borne viral infection which predominately affects sheep causing neurological disease. Louping ill may also affect goats and cattle. The condition has been reported in humans and is classified as a zoonosis [1]. Louping ill is considered a significant problem among red grouse in Scotland [2]. Louping ill is caused by louping ill virus which is a flavivirus [1]. The name 'louping-ill' is derived from an old Scottish word describing the effect of the disease in sheep whereby they 'loup' or spring into the air. Louping ill gives rise to a diffuse non-suppurative meningoencephalitis and manifests clinically with ataxia, pyrexia, seizures and opisthotonous, posterior paralysis, coma and death [3]. The disease is also associated with sudden death. Concurrent tick borne fever infection (*Anaplasma phagocytophilum*) is thought to amplify the effect of the louping ill virus [4]. Morbidity rates of between five and 60% have been reported [3]. The condition is transmitted by the tick *Ixodes ricinus*, and its prevalence is closely associated with the distribution of this vector [5]. There is a seasonal occurrence in the spring and autumn, coinciding with the activity of the tick vector [3]. The presence of deer is associated with an increased tick population in a locality [5]. Climate change has been implicated in increased tick populations [5].

The objective of this study was to estimate the prevalence of exposure to louping ill virus among carcasses submitted for routine post mortem examination to Sligo Regional Veterinary Laboratory, in the North West of Ireland. There have been no previous studies of the prevalence of louping ill in Ireland.

## Methods

Blood samples were collected from 199 sheep from 152 flocks, which had been submitted to Sligo RVL during 2011. The samples were collected opportunistically at routine post-mortem examination. Carcasses are accepted for post mortem examination on the basis that the herd owner consents to any tests carried out at the discretion of the veterinary pathologist. Sera were tested using a goose blood haemagglutination inhibition assay at the Virology Section in the Moredun Research Institute, Scotland. Samples were titrated out in the presence of a constant virus antigen and goose red blood cells. The first dilution was 1:10 with doubling dilutions thereafter. A titre of 1:20 was used as the cut off for positive / negative results. Titres were expressed as the highest dilution at which inhibition of haemagglunination was detected. A presumptive diagnosis of louping ill was made based on histopathological changes associated with non-suppurative meningoencephalitis in the brains of affected animals consistent with a diagnosis of louping ill.

**Figure 1:**
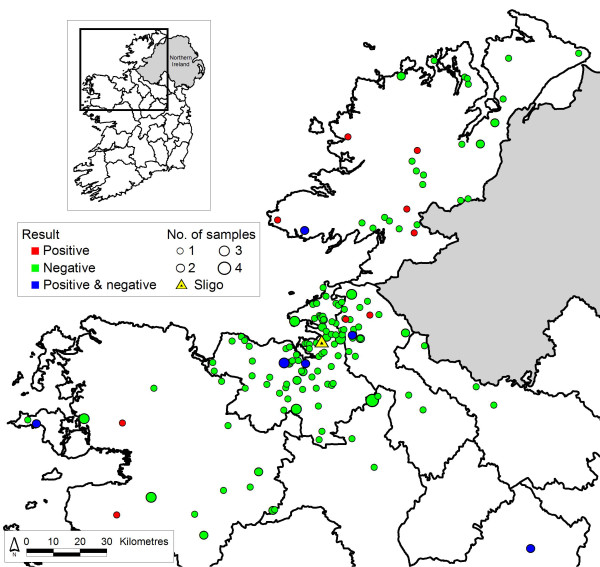
Shows the location of sampled sheep flocks.

Maps were created using ESRI Arcview 3.2 software (Redlands; California). Flocks were represented by the centroid of the largest fragment of land associated with each flock, or the corresponding townland where a flock number was not available (2 flocks) (Figure 1).

For the elevation analysis, the centroid of all fragments in a farm was calculated and used to create a point map. This point map was then queried to find the intersection value of an underlying digital elevation model surface using a bilinear interpolation of each value (ArcGIS 10, ESRI, Redlands, CA, USA). This addresses the issue of a value from a single point representing a fragment of land. The output consisted of an elevation value in metres above sea level for all fragments of land in the study.

## Results

Seventeen samples showed serological evidence of exposure to louping ill virus from the 199 blood samples, giving an animal level prevalence of 8.5%. These positive samples came from 15 of the 152 flocks, giving a flock level prevalence of 9.8%. Two flocks each had two sero-positive animals, while the remaining 13 flocks had a single sero-positive animal. Six flocks had both positive and negative samples. The greatest proportion of sero-positive animals was found among the adult age group (Table 1). The temporal distribution of the detection of sero-positive animals is presented in Table 2. The geographical distribution of positive cases is outlined in Table 3. The greatest number of cases was in Co. Donegal. The post mortem diagnosis and the signalment of the 17 sero-positive animals are presented in Tables 4 and 5 respectively. There was a difference of 106 metres in the average maximum elevation of land parcels of holdings associated with positive flocks grazed was 106 metres higher than that the maximum elevation of the land associated with negative flocks (Table 6), and there was a 40 metre difference in the mean elevation between the positive and negative flocks.

**Table 1:** Age breakdown of louping ill positive sheep

**Age category**	**No positive**	**No submitted**	**Prevalence(%)**
0-6 month old	0	43	0
6-12 month old	2	35	5.7
12-24 month old	0	17	0
Greater than 24 months	15	104	14.4
Total	17	199	8.5

**Table 2:** Month of submission of louping ill positive sheep

**Month submitted**	**No.positive**	**No submitted**	**% seropositive**
January 2011	0	14	0
February 2011	1	7	14.3
March 2011	2	37	5.4
April 2011	5	35	14.2
May 2011	0	16	0
June 2011	1	12	8.3
July 2011	1	6	16.6
August 2011	1	10	10
September 2011	1	17	5.8
October 2011	1	21	4.8
November 2011	1	6	16.6
December 2011	3	18	16.6

**Table 3:** Submission of samples by county

**County**	**No.positive**	**No submitted**	**% seropositive**
Cavan	0	6	0
Donegal	8	38	21.0
Leitrim	3	27	11.1
Longford	1	2	-
Mayo	3	23	13.0
Roscommon	0	9	0
Sligo	2	94	2.1

**Table 4:** Post mortem diagnosis of 17 seropositive samples

**Diagnosis**	**No.of cases**
Louping ill	3
Listeriosis	3
No diagnosis	1
Septicaemia	3
Pneumonia	3
Pregnancy toxaemia (twin lamb disease)	2
Parasitic gastro enteritis	1
Acute Fascioliasis	1

**Table 5:** Signalment of 17 seropositive cases

**History**	**No.of cases**
Pneumonia / tachypnoea	2
Ataxia	5
Ataxia and / tachypnoea	2
Sudden death	4
Dull	2
Lameness	1
Ill thrift	1

**Table 6:** Comparison of the mean and median of the maximum, minimum and mean elevations for flocks where antibodies to louping ill were detected to flocks where no antibodies were detected

		**Positive flocks**	**Negative flocks**	**Difference**
Max elevation (m)	Mean	277.0	170.8	106.2
	Median	254.0	113.5	140.5
Min Elevation (m)	Mean	59.7	52.0	7.7
	Median	56.5	47.0	9.5
Mean Elevation (m)	Mean	134.5	94.3	40.2
	Median	105.0	83.5	21.5

## Discussion and evaluation

This study has found an animal level seroprevalence of 8.5% and a flock level seroprevalence of 9.8%.

However, caution would be advised in extrapolating these figures to the national sheep population as the sample is biased due to the opportunistic nature of the sampling protocol. Secondly, a greater proportion than the national average of the land area within the Sligo Regional Veterinary Laboratory (RVL) catchment area would be classified as upland with rough grazing, and therefore would be more likely to have a tick burden, than areas with a greater proportion of improved grassland. Further there was an uneven distribution of submissions from the counties within the catchment area. It has been shown in a previous report that a significant determinant in submitting a carcass to an RVL is distance from the submitting farm to the RVL [6] and this study emphasises this further with 47% of submissions coming from Co. Sligo. The relatively small sample size and the number of positive animals may also raise concerns relating to study power. Despite these shortcomings, this study provides interesting information on the epidemiology of louping ill in Irish sheep flocks in the northwest of Ireland.

The greatest proportion of sero-positive animals was in the adult (greater than 24 month old) category. This may in part reflect historical seroconversion where an animal developed immunity following infection, and may not be necessarily associated with active infection at the time of sampling.

If an adult sheep were introduced to the farmland, it would have had no prior immunity to the condition and would be more susceptible. Of the 17 cases encountered in the present study, three of these were in recently purchased sheep. However, it cannot be determined if these affected sheep had been on a tick-infested land parcel earlier in their lives. Traditionally, a seasonal pattern of occurrence has been observed with louping ill [3]. Louping ill and listeriosis were diagnosed at post mortem in six of the 17 positive cases.

The elevation of the land grazed by sheep in flocks where louping ill was diagnosed was greater than that of the flocks where no sero-positive animals were found. This is as expected as the tick required for the transmission of louping ill is found on rough grazing land which tends to be at higher elevations.

Despite the awareness of louping ill as an endemic disease in Ireland for many years, this is the first published report detailing its occurrence in the north west of Ireland.

## Conclusion

There was an animal level prevalence of 8.5%, and a flock level prevalence of 9.8%. The greatest proportion of sero-positive animals was identified among the animals older than 24 months of age. The elevation of the land associated with positive flocks was greater than that of negative flocks.

## Competing interests

The authors declare that they have no competing interest.

## Authors’ contributions

DB collected samples, did the data analysis and wrote the paper. CÓM collected samples, organised the logistics of testing and reviewed the paper. DMC carried out the GIS analysis and reviewed the paper. GMcG carried out the elevation analysis and reviewed the paper. All authors read and approved the final manuscript.
